# Molecular Targets for Breast Cancer Therapy

**DOI:** 10.3390/biom14101219

**Published:** 2024-09-27

**Authors:** Hamidreza Montazeri Aliabadi

**Affiliations:** Department of Biomedical and Pharmaceutical Sciences, Chapman University School of Pharmacy, Harry and Diane Rinker Health Science Campus, Irvine, CA 92618, USA; montazer@chapman.edu

Breast cancer is by far the most common cancer in women, and for a while, it surpassed lung cancer as the most diagnosed cancer, regardless of gender, in 2020 [1]. Chemotherapy and hormone therapy are still the first line of treatment, despite extensive research in molecularly targeted drugs. Therefore, triple negative breast cancer (TNBC) is still known as the most challenging type for treatment due to the limited identified targets. Inherent and acquired resistance are still major hurdles in breast cancer therapy, which further highlights the importance of identifying new molecular targets in this battle. The inherent resistance of unresponsive cells could be explained by tumor heterogeneity. In a “Big Bang” model proposed in 2015, a “spatial heterogeneity” is illustrated due to consecutive mutations in different generations of cancer cells within a single tumor [2]. This heterogeneity has been demonstrated in a study of two human acute lymphoblastic leukemia samples using viSNE technology, which shows that the differences between the samples outnumber the similarities [3]. Obviously, this intra-tumoral heterogeneity also affects the theory of personalizing the treatment based on the biomarker expression in a specific patient. Even if the selected molecularly targeted drug eradicates all the “sensitive” cells that rely on the targeted molecule for their survival, a sub-population of the population of cells would survive and trigger a relapse [4]. This “Darwinian” model is reported in a variety of cancer types [5]. On the other hand, the plasticity of cancer cells that enables adapting to molecularly targeted drugs could explain the acquired resistance. In addition to point mutations, the availability of a variety of pathways leading to enhanced proliferation and survival could be partially responsible for the intracellular adjustments required. We have previously reported the possibility of overcoming inherent or acquired resistance to molecularly targeted drugs by targeting well-selected alternative proteins. This Special Issue aimed to collect current efforts and insights into the most recent developments in potential molecular targets in breast cancer therapy. The Special Issue presents three original research articles and two review articles.

Abbasi Dezfouli et al. report a nanoparticle design to deliver small interfering RNAs (siRNAs) to the triple-negative breast cancer (TNBC) cell line, MDA-MB-231, and demonstrate the efficiency of this approach in silencing the expression of survivin in this cell line [6]. The overactivation of anti-apoptosis pathways and/or inhibition of pro-apoptosis procedures are among the mechanisms involved in carcinogenesis [7], cancer progression [8], and/or resistance against anticancer treatments [9,10,11]. Among the anti-apoptosis proteins, survivin, a member of “inhibitors of apoptosis proteins (IAP), is reported to be involved in different mechanisms, which all result in cell survival [12]. Survivin is overexpressed in a variety of cancer types, including breast cancer [13], but is indetectable in normal cells [14]. Dr. Uludag’s group (the corresponding author of this manuscript) has been working for quite some time on hydrophobically modified low molecular weights for complex formation and delivery of siRNAs to different cancer cells; however, in this paper, they report the effect of adding an anionic component to the nanoparticles on the internalization of siRNA and silencing efficiency [6].

Vaganova et al. manuscript focuses on trace amine-associated receptors (TAARs) and their involvement in breast cancer progression [15]. It has been previously reported that TAAR1 expression enhances the positive survival rate in breast cancer patients [16]. However, in a more recent study, Vaganova et al. have revealed an association between TAAR6 and the mTOR pathway that suggested TAARs’ role in melanoma pathogenesis [17]. In the present study, authors analyze publicly available transcriptomic data to estimate TAARs’ association with breast cancer subtypes. They evaluated the association between the TAAR family members’ expression (TAAR1, TAAR2, TAAR5, TAAR6, TAAR8, and TAAR9) and the stage, grade, and subtype of the breast cancer patient. They reported that TAAR expression was upregulated in basal-like and HER2-positive sub-types as well as in circulating tumor cells (as compared to metastatic lesions). The authors concluded that TAAR1 and perhaps other members of the TAAR family could be further studied as potential targets in breast cancer treatment due to the co-expression of these receptors and G-protein-coupled receptors (GPCRs) [15].

Metal–organic frameworks (MOFs) have been extensively evaluated as delivery systems for anticancer drugs in the last decade. They have been reported as hybrid porous materials that are built from metal ions or clusters bridged by organic linkers [18]. They offer adjustable porosity, a large surface area, biocompatibility, and the possibility to functionalize for targeted delivery, which makes them promising candidates for delivery of anticancer drugs [19]. MOF-based delivery systems have been used in targeted delivery to breast cancer cells for different purposes, including delivering gene silencing tools, DNAzymes, and rapamycin to TNBC cells in vivo [20], nanocarriers for magnetic resonance imaging of 4T1-Luc breast tumors in a mouse model [21], delivering honokiol and indocyanine green for enhancing photochemotherapy of breast tumors [22], and tumor microenvironment remodeling in 4T1 tumor-bearing mice [23]. The paper by Rajamohan et al. in this Special Issue reports encapsulation of adenosine (AND) in β-cyclodextrin and potassium ions using a diffusion method. The authors use an approach based on quantum mechanics to investigate the formation of hydrogen bonds and energy parameters to better understand the interactions between the components of the MOFs. They also evaluate the cytotoxicity of these MOFs in vitro in the MDA-MB-231 TNBC cell line [24].

Targeted therapies for TNBC are the topic of Chapdelaine and Sun’s review paper included in this Special Issue [25]. The authors emphasize the heterogeneity in molecular drivers of TNBC, which they suggest would require combinatorial therapies to target more than one molecular driver at the same time. The manuscript reviews some of the FDA-approved molecularly targeted drugs and then covers some of the ongoing clinical trials targeting phosphatidylinositol-3 kinase (PI3K), epithelial growth factor receptor (EGFR), Src, and Mitogen-activated protein kinases (MAPKs) pathways, and then discusses combination treatments in the clinical trial stage. However, they correctly point out that most of these efforts combine chemotherapy with a molecularly targeted drug, except for a clinical trial combining GSK214179, an AKT inhibitor, with trametinib as a MEK inhibitor (NCT0196392), which showed an improvement in clinical benefit rate for the combination over trametinib monotherapy. The authors then review mathematical modeling approaches that could result in the identification of promising combinations of molecularly targeted drugs and conclude with some of the new treatment strategies, including drugs targeting metabolism in cancer cells or epigenic therapy [25].

Finally, in a review paper by our research group, we reviewed all the molecularly targeted FDA-approved drugs for breast cancer treatment (including hormone-related molecules) [26]. A simple look at the 23 drugs included in this list (as of 10 February 2023), 35% were targeting hormone-related molecules, and 26% target a member of the HER family of receptors, for a total of 61% (14 out of 23). Since that date, the FDA has approved capivasertib (AZD5363), an AKT inhibitor, to be used along with fulvestrant, an estrogen receptor inhibitor, in adult patients with hormone receptor-positive, HER2-negative locally advanced or metastatic breast cancer with PI3K/AKT mutation(s) (16 November 2023) [27]. However, despite this new entry and the other molecularly targeted drugs previously approved, it seems that we are still heavily relying on hormone (or hormone-related) therapy and HER2 targeting. In the manuscript, after reviewing the “familiar” (hormone therapy and HER2 and CDK4/6 targeting drugs), we reviewed some of the emerging strategies in molecular targets in breast cancer treatment, which include immune checkpoint inhibitors, PARP inhibitors, and PI3K/AKT pathway inhibitors. Among these strategies, targeting immune checkpoints (also referred to as immunotherapy) has been extensively studied in different cancer types and seems promising. PD-1/PD-L1 pairs on T cells/cancer cells seem to be the most popular targets, which we thought could be at least partially since PD-1 was the first target discovered among the checkpoints. We also noticed that targeting the checkpoint expressed on the T cells is more attractive to clinicians as compared to the counterpart expressed on the tumor cells, which we again proposed to be explained (at least partially) to the immune cells being more “accessible”. The last part of this manuscript reviews the signaling pathways and molecular targets that have not been clinically used despite extensive research. Among the targets reviewed in this section are Src, RAS/RAF/MEK/ERK pathway, JAK/STAT pathway (which we have reviewed previously as well [28]), and other immerging targets, e.g., peroxisome proliferator-activated receptors (PPARs), and novel strategies such as chimeric antigen receptor (CAR) T-cell immunotherapy.

We have come a long way in studying and understanding the complicated signaling pathways that enable cancer cells to proliferate and survive the harshest biological conditions and chemotherapy assaults; however, the crosstalk among these interconnected pathways has proven to be an obstacle difficult to overcome with single therapy. While combinatorial therapy seems to be a major part of ongoing clinical trials, it is surprising that most of the combinations are exploring chemotherapy/molecular targeting instead of two or more molecular targets that take part in those intercellular crosstalks. We hope that this Special Issue will trigger some thoughts on the new directions that could be explored to expand molecular targeting as a major component of clinical breast cancer treatment.

## References

[1] Arnold M., Morgan E., Rumgay H., Mafra A., Singh D., Laversanne M., Vignat J., Gralow J.R., Cardoso F., Siesling S. (2022). Current and future burden of breast cancer: Global statistics for 2020 and 2040. Breast.

[2] Sottoriva A., Kang H., Ma Z., Graham T.A., Salomon M.P., Zhao J., Marjoram P., Siegmund K., Press M.F., Shibata D. (2015). A Big Bang model of human colorectal tumor growth. Nat. Genet..

[3] Amir E.-A.D., Davis K.L., Tadmor M.D., Simonds E.F., Levine J.H., Bendall S.C., Shenfeld D.K., Krishnaswamy S., Nolan G.P., Pe’er D. (2013). viSNE enables visualization of high dimensional single-cell data and reveals phenotypic heterogeneity of leukemia. Nat. Biotechnol..

[4] Alderton G.K. (2013). Tumour heterogeneity: The rise of the minority. Nat. Rev. Cancer.

[5] Holzel M., Bovier A., Tuting T. (2013). Plasticity of tumour and immune cells: A source of heterogeneity and a cause for therapy resistance?. Nat. Rev. Cancer.

[6] Abbasi Dezfouli S., Rajendran A.P., Claerhout J., Uludag H. (2023). Designing Nanomedicines for Breast Cancer Therapy. Biomolecules.

[7] Chaudhry G.E., Md Akim A., Sung Y.Y., Sifzizul T.M.T. (2022). Cancer and apoptosis: The apoptotic activity of plant and marine natural products and their potential as targeted cancer therapeutics. Front. Pharmacol..

[8] Pfeffer C.M., Singh A.T.K. (2018). Apoptosis: A Target for Anticancer Therapy. Int. J. Mol. Sci..

[9] Ling V.Y., Straube J., Godfrey W., Haldar R., Janardhanan Y., Cooper L., Bruedigam C., Cooper E., Tavakoli Shirazi P., Jacquelin S. (2023). Targeting cell cycle and apoptosis to overcome chemotherapy resistance in acute myeloid leukemia. Leukemia.

[10] Shahar N., Larisch S. (2020). Inhibiting the inhibitors: Targeting anti-apoptotic proteins in cancer and therapy resistance. Drug Resist. Updat..

[11] Tian X., Srinivasan P.R., Tajiknia V., Sanchez Sevilla Uruchurtu A.F., Seyhan A.A., Carneiro B.A., De La Cruz A., Pinho-Schwermann M., George A., Zhao S. (2024). Targeting apoptotic pathways for cancer therapy. J. Clin. Investig..

[12] Yamamoto H., Ngan C.Y., Monden M. (2008). Cancer cells survive with survivin. Cancer Sci..

[13] Jha K., Shukla M., Pandey M. (2012). Survivin expression and targeting in breast cancer. Surg. Oncol..

[14] Parmar M.B., K C R.B., Lobenberg R., Uludag H. (2018). Additive Polyplexes to Undertake siRNA Therapy against CDC20 and Survivin in Breast Cancer Cells. Biomacromolecules.

[15] Vaganova A.N., Maslennikova D.D., Konstantinova V.V., Kanov E.V., Gainetdinov R.R. (2023). The Expression of Trace Amine-Associated Receptors (TAARs) in Breast Cancer Is Coincident with the Expression of Neuroactive Ligand-Receptor Systems and Depends on Tumor Intrinsic Subtype. Biomolecules.

[16] Vattai A., Akyol E., Kuhn C., Hofmann S., Heidegger H., von Koch F., Hermelink K., Wuerstlein R., Harbeck N., Mayr D. (2017). Increased trace amine-associated receptor 1 (TAAR1) expression is associated with a positive survival rate in patients with breast cancer. J. Cancer Res. Clin. Oncol..

[17] Vaganova A.N., Kuvarzin S.R., Sycheva A.M., Gainetdinov R.R. (2022). Deregulation of Trace Amine-Associated Receptors (TAAR) Expression and Signaling Mode in Melanoma. Biomolecules.

[18] Wu M.X., Yang Y.W. (2017). Metal-Organic Framework (MOF)-Based Drug/Cargo Delivery and Cancer Therapy. Adv. Mater..

[19] Coluccia M., Parisse V., Guglielmi P., Giannini G., Secci D. (2022). Metal-organic frameworks (MOFs) as biomolecules drug delivery systems for anticancer purposes. Eur. J. Med. Chem..

[20] Liu P., Liu X., Cheng Y., Zhong S., Shi X., Wang S., Liu M., Ding J., Zhou W. (2020). Core-Shell Nanosystems for Self-Activated Drug-Gene Combinations against Triple-Negative Breast Cancer. ACS Appl. Mater. Interfaces..

[21] Meng Z., Huang H., Huang D., Zhang F., Mi P. (2021). Functional metal-organic framework-based nanocarriers for accurate magnetic resonance imaging and effective eradication of breast tumor and lung metastasis. J. Colloid. Interface. Sci..

[22] He Y., Guo J., Ding H., Lin M., Wu Y., He Z., Wang Z., Xia Q., Zhu C., Zhang Y. (2024). Glutathione-responsive CD-MOFs co-loading honokiol and indocyanine green biomimetic active targeting to enhance photochemotherapy for breast cancer. Int. J. Pharm..

[23] Wu H., Jin M., Liu Y., Wang S., Liu C., Quan X., Jin M., Gao Z., Jin Y. (2024). A self-targeting MOFs nanoplatform for treating metastatic triple-negative breast cancer through tumor microenvironment remodeling and chemotherapy potentiation. Int. J. Pharm..

[24] Rajamohan R., Ashokkumar S., Murali Krishnan M., Murugavel K., Murugan M., Lee Y.R. (2023). Adenosine/beta-Cyclodextrin-Based Metal-Organic Frameworks as a Potential Material for Cancer Therapy. Biomolecules.

[25] Chapdelaine A.G., Sun G. (2023). Challenges and Opportunities in Developing Targeted Therapies for Triple Negative Breast Cancer. Biomolecules.

[26] Montazeri Aliabadi H., Manda A., Sidgal R., Chung C. (2023). Targeting Breast Cancer: The Familiar, the Emerging, and the Uncharted Territories. Biomolecules.

[27] Mullard A. (2024). FDA approves first-in-class AKT inhibitor. Nat. Rev. Drug. Discov..

[28] Bousoik E., Montazeri Aliabadi H. (2018). “Do We Know Jack” About JAK? A Closer Look at JAK/STAT Signaling Pathway. Front. Oncol..

